# E50K-OPTN-Induced Retinal Cell Death Involves the Rab GTPase-Activating Protein, TBC1D17 Mediated Block in Autophagy

**DOI:** 10.1371/journal.pone.0095758

**Published:** 2014-04-21

**Authors:** Madhavi Latha Somaraju Chalasani, Asha Kumari, Vegesna Radha, Ghanshyam Swarup

**Affiliations:** Centre for Cellular and Molecular Biology, Council of Scientific and Industrial Research, Hyderabad, India; National Institute of Biological Sciences, Beijing, China

## Abstract

The protein optineurin coded by *OPTN* gene is involved in several functions including regulation of endocytic trafficking, autophagy and signal transduction. Certain missense mutations in the gene *OPTN* cause normal tension glaucoma. A glaucoma-causing mutant of optineurin, E50K, induces death selectively in retinal cells. This mutant induces defective endocytic recycling of transferrin receptor by causing inactivation of Rab8 mediated by the GTPase-activating protein, TBC1D17. Here, we have explored the mechanism of E50K-induced cell death. E50K-OPTN-induced cell death was inhibited by co-expression of a catalytically inactive mutant of TBC1D17 and also by shRNA mediated knockdown of TBC1D17. Endogenous TBC1D17 colocalized with E50K-OPTN in vesicular structures. Co-expression of transferrin receptor partially protected against E50K-induced cell death. Overexpression of the E50K-OPTN but not WT-OPTN inhibited autophagy flux. Treatment of cells with rapamycin, an inducer of autophagy, reduced E50K-OPTN-induced cell death. An LC3-binding-defective mutant of E50K-OPTN showed reduced cell death, further suggesting the involvement of autophagy. TBC1D17 localized to autophagosomes and inhibited autophagy flux dependent on its catalytic activity. Knockdown of TBC1D17 rescued cells from E50K-mediated inhibition of autophagy flux. Overall, our results suggest that E50K mutant induced death of retinal cells involves impaired autophagy as well as impaired transferrin receptor function. TBC1D17, a GTPase-activating protein for Rab GTPases, plays a crucial role in E50K-induced impaired autophagy and cell death.

## Introduction

Glaucoma is a heterogeneous group of optic neuropathies characterized by the death of retinal ganglion cells and its axons leading to permanent blindness [1], [2]. High intraocular pressure is a major risk factor but not always sufficient to cause the neuropathy. Multiple genetic and environmental factors play a major role in glaucoma etiology. More than 20 genetic loci have been linked to primary open angle glaucoma (POAG), which is the major type of disease, but only a few genes have been identified, including *MYOC, OPTN* and *WDR36* [3], [4]. Mutations in *OPTN* are associated mainly with normal tension glaucoma, a subset of POAG, where intraocular pressure is within normal limits (10–20mm Hg) but retinal ganglion cell death is observed leading to glaucoma [5]. Later on, certain mutations in *OPTN* were shown to cause amyotrophic lateral sclerosis [6]. Optineurin is localized to pathological structures seen in several neurodegenerative diseases such as amyotrophic lateral sclerosis, Alzheimer’s disease, Parkinson’s disease, etc [6], [7].

The gene, *OPTN*, codes for the protein optineurin, which is involved in a variety of functions including regulation of endocytic trafficking, autophagy, immune response, mitosis and NF-κB signal transduction [8]–[19]. Many missense mutations in optineurin have been reported and the association of the mutation with the disease varies in different populations [5], [20]–[25]. However, functional alterations caused by mutations in optineurin are poorly understood.

The mutant E50K, where glutamic acid at position 50 is mutated to lysine, is a severe disease causing mutation which is located in the conserved bZIP region [5]. This mutant induces cell death selectively in retinal cells but not in other cell lines tested which include IMR-32, Hela and Cos-1 [26]. Studies carried out by Chi *et al* have further revealed that E50K transgenic mice show severe retinal degeneration where all the retinal cell layers are affected [27]. This mutant causes defective endocytic trafficking and recycling of transferrin receptor (TFR) resulting in the formation of large vesicle-like structures or foci positive for transferrin receptor [8], [28]. E50K mutant shows altered interaction with TBK1 [29], [30].

It has been suggested that E50K-induced death of retinal cells involves autophagy, a quality control mechanism that is used by the cells to remove damaged proteins and organelles through lysosomal degradation [31], [32]. Autophagy is basically a membrane vesicle trafficking event which involves formation of autophagosomes that sequester damaged and aggregated proteins, and damaged organelles for degradation. The autophagosomes fuse with lysosomes to form autolysosomes where degradation of macromolecules occurs [32], [33]. Some of the Rab GTPases are involved in autophagy [34]. The activity of Rab GTPases, which control almost all the steps involved in vesicle trafficking, is regulated by guanine nucleotide exchange factors that activate them, and GTPase-activating proteins (GAPs), which inactivate them by converting from active, GTP-bound state to inactive, GDP-bound state.

TBC1D17, a GAP for Rab GTPases, was identified as an optineurin-interacting protein in a yeast two-hybrid screen for novel optineurin-interacting proteins [35]. *In vitro,* it acts on several Rabs, but in the cells it acts on Rab8 to regulate endocytic trafficking of TFR [36], [37]. Regulation of Rab8 activity and function by TBC1D17 is mediated by optineurin which also mediates interaction of Rab8 with TBC1D17 [37]. The E50K mutant causes defective endocytic recycling of TFR that is mediated by TBC1D17-dependent inactivation of Rab8 [37].

Here, we have explored the role of TBC1D17 and autophagy in E50K-induced cell death. For this purpose, we have used a retinal cell line, earlier known as retinal ganglion cell line RGC-5 which was the only ganglion cell line available for studies pertaining to glaucoma [38]. This cell line has been re characterized and identified as similar to a mouse retinal photoreceptor cell line [39]. This cell line shows properties of neuronal precursor cells [38]. Although, it is not a retinal ganglion cell line it is still a useful cell culture model to study mechanisms associated with E50K-optineurin induced cell death relevant for glaucoma due to the following reasons: (a) In glaucoma, in addition to ganglion cells in the inner retina, outer retinal cells such as photoreceptor cells (cone cells) are also damaged in experimental animal models as well as humans [40]–[45],; (b) In transgenic mice expressing E50K-optineurin all the cell layers in retina are affected leading to reduced thickness of retina due to cell death [27]. In addition, E50K mutant induces cell death in this cell line selectively but not in many other cell lines tested [26].

## Materials and Methods

### Plasmids and Reagents

Plasmid vectors HA-OPTN/E50K, GFP-OPTN/E50K and the double mutant, E50K-D474N have been described earlier [8], [26]. GFP-E50K-F178A was made by site-directed mutagenesis using GFP-E50K as template. The primers used for constructing the F178A mutant of E50K were as follows: Forward primer- 5′-GGCTCCTCAGAAGATTCCGCTGTTGAAATTAGGATGGC-3′. Reverse primer- 5′-GCCATCCTAATTTCAACAGCGGAATCTTCTGAGGAGCC-3′. Human TBC1D17 expression plasmids with HA and GFP tags have been described previously [37]. Human TFR expression plasmid and mouse Rab12 with HA and GFP tags have been described previously [10]. Plasmids, GFP-LC3B and mCherry-GFP-LC3B were kindly provided by Dr. Terje Johansen (University of Tromsø, Tromsø, Norway).

### Antibodies

Mouse monoclonal and rabbit polyclonal HA, mouse monoclonal GFP and rabbit polyclonal cdk2 antibodies were from Santa Cruz Biotechnology. Rabbit polyclonal cleaved caspase-3 antibody was from Cell Signaling Technology. Mouse monoclonal TFR antibody and rabbit polyclonal TBC1D17 antibody were obtained from Invitrogen. Rabbit polyclonal p62/SQSTM1 antibody was from Sigma-Aldrich and mouse monoclonal LC3 antibody was from Enzo Life Sciences. Secondary antibodies Cy-3 conjugated anti-mouse and anti-rabbit IgG, and HRP conjugated anti-mouse and anti-rabbit IgGs were from Amersham. Alexa-488 and Alexa-633 conjugated anti-mouse and anti-rabbit IgGs were from Molecular Probes.

### ShRNA Vectors for Mouse TBC1D17

ShRNA vectors targeting mouse TBC1D17 (shRNA1 and shRNA2) were constructed using pmU6 vector, which is an U6-promoter based vector as explained previously [46], [47]. The mouse TBC1D17 sequence targeted by shRNA1 was from 1256 to 1274 and the sequence targeted by shRNA2 was from 1243 to 1261 (GenBank accession. NM_ 001042655.1). A vector expressing shRNA of unrelated sequence (ConshRNA) of the same length was used as a control.

### Cell Culture and Transfections

All the experiments were carried out using a cell line previously reported as retinal ganglion cell line, RGC-5 [38] but recently shown to be the same or very similar to a mouse photoreceptor cell line, 661W [39]. These cells were grown as monolayers in a humidified atmosphere of 5% CO_2_ at 37°C in Dulbecco’s Modified Eagle’s Medium (DMEM) containing 10% fetal calf serum, 100 U/ml penicillin and 100 µg/ml streptomycin. Transient transfections in these cells were performed using Qiagen column (Qiagen, Valencia, CA) purified plasmids and Lipofectamine 2000 (Invitrogen, San Diego, CA, USA) reagent according to the manufacturer’s instructions.

### Indirect Immunofluorescence and Confocal Microscopy

The cells grown on coverslips were transfected with required plasmids and allowed to overexpress proteins for the required time. The cells were then fixed and indirect immunofluorescence of cells was carried out as described previously [48].

To study colocalization, stained coverslips were observed under LSM 510 NLO Meta Confocal Microscope (Carl Zeiss) and images were captured using 63X/1.4 NA oil immersion objective. For imaging purpose, a 488 nm argon laser, 561 nm DPSS laser and 633 nm HeNe633 laser were used for GFP, Cy3 and Alexa633 respectively. Colocalization was determined by observing the staining pattern and two middle sections were projected to observe colocalization using LSM 510 (version 3.2) software. Colocalization was quantitated by calculating Pearson’s correlation coefficients using the LSM 510 software.

### Cell Death Assays

Quantitative analysis of cell death was carried out essentially as described earlier [26], [49]. Cells plated on coverslips were either transfected with E50K-OPTN alone or cotransfected with required amount of other plasmids and were allowed to overexpress for 32 hours. The cells were then fixed and stained with HA antibody and mounted with mountant containing DAPI which stains DNA. The cells showing fluorescence were counted and taken as expressing cells and those cells which showed loss of refractility, chromatin condensation and cell shrinkage were scored as apoptotic or dead cells. At least 200 expressing cells were counted from each coverslip to determine the percentage of cell death. Cells not expressing the transfected proteins (non-expressing cells) were also counted from each coverslip and cell death in these cells was 1 to 3%.

### Autophagy Flux Assays

To induce autophagy, cells were incubated with amino acid and serum-free media (EBSS or Earle’s balanced salt solution) for 2 hours. To study autophagy flux, a reporter construct mCherry-GFP-LC3B was co expressed with the required plasmids and images taken using LSM 510 Meta NLO Confocal Microscope with 63X oil immersion objective lens (NA 1.4). Optical Z-sections of 0.33 µm were taken from top to the bottom of the cell. These images were then analyzed using Imaris software (Bitplane, Switzerland) for measuring total number of green dots (or yellow dots/autophagosomes) and total number of red dots. Only red dots, which represent autolysosomes were calculated by subtracting green/yellow dots from total red dots for each cell.

### SDS-PAGE and Western Blotting

The proteins were resolved by SDS-PAGE and transferred to nitrocellulose membrane (Hybond-ECL from Amersham) using semi-dry transfer apparatus (Amersham). Western blot analysis was done as described previously [48]. The blots were quantitated using ImageJ software.

### Statistical Analysis

Statistical significance of differences among experimental groups was assessed by Student’s *t* test. *P* values less than 0.05 were considered statistically significant. The level of significance was shown at 3 levels, viz., *0.05, **0.01 and ***0.001.

## Results

### TBC1D17 Mediates E50K-induced Death in Retinal Cells

**Figure 1:**
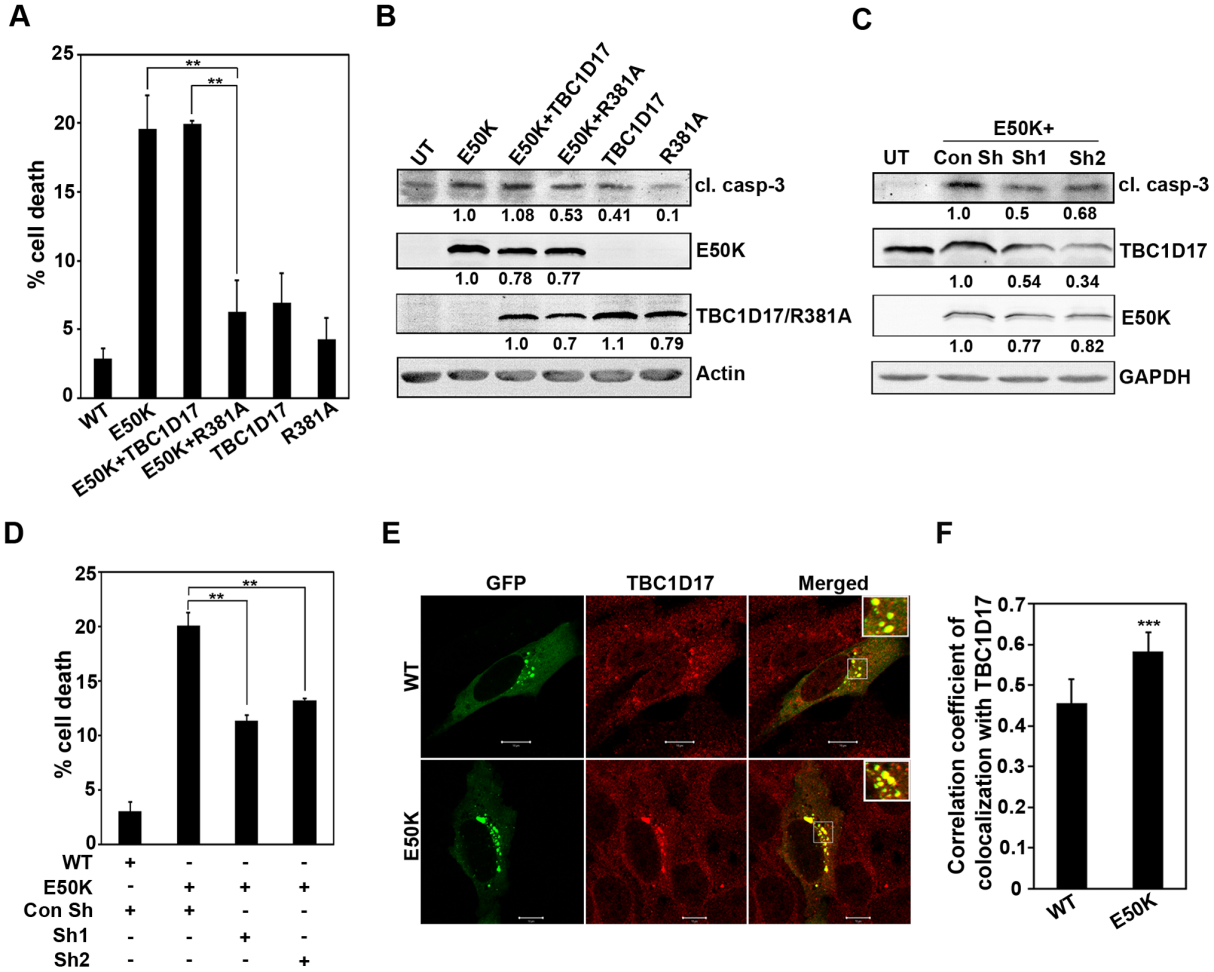
E50K-OPTN-induced cell death is dependent on TBC1D17. (A) Cells plated on coverslips were transfected with HA-E50K alone or cotransfected with HA-E50K and GFP-TBC1D17 or GFP-R381A. After 32 hours of overexpression, cells were fixed and stained with HA antibody. Bar diagram depicts percentage of cells showing E50K-induced death in the presence of TBC1D17 and R381A after subtracting background cell death in non-expressing cells. When coexpressing E50K and TBC1D17 or R381A, cells expressing both E50K and TBC1D17/R381A were counted as expressing cells. Data represent mean ± SD of 4 experiments. **p<0.01 (Student’s t-test) (B) Cells plated in 35-mm dishes were transfected with GFP-E50K alone or cotransfected with GFP-E50K and HA-TBC1D17 or R381A. Protein lysates were made at the end of 32 hours of overexpression and subjected to western blotting with cleaved caspase-3 (cl. casp-3), GFP and HA antibodies. Actin was used as a loading control. UT, untransfected. The numbers below the blots indicate relative level of expression normalized with the loading control. (C) Cells plated in 35-mm dishes were cotransfected with 1 µg of GFP-E50K and 2 µg of control shRNA (Con Sh) or shRNAs against mouse TBC1D17 (sh1, sh2). Cell lysates were made at the end of 32 hours of overexpression and subjected to western blotting with cleaved caspase-3, GFP and TBC1D17 antibodies. GAPDH was used as a loading control. (D) Cells plated on coverslips were transfected with 150ng of GFP-E50K along with 300ng of control shRNA plasmid or TBC1D17 shRNA plasmids (sh1, sh2). After 32 hours of transfection, cells were fixed and cell death was determined in E50K expressing cells. Bar diagram depicts percentage of cells showing E50K-induced death in the presence of control shRNA and shRNA against TBC1D17 after subtracting background cell death in non-expressing cells. Data represent mean ± SD of 3 experiments. **p<0.01 (Student’s t-test) (E) Cells were transfected with 100ng of GFP-WT OPTN or GFP-E50K. After 24 hours of transfection, cells were fixed, stained with TBC1D17 antibody and analysed by confocal microscopy. Colocalization is shown by merging of red (TBC1D17) with green (WT/E50K) giving yellow color in the merged images. Scale bar: 10 µm. (F) Bar diagram showing correlation coefficient of colocalization of endogenous TBC1D17 (red) with WT-OPTN or E50K (green). n = 15 cells. ***p<0.001.

E50K mutant of optineurin causes death in retinal cells but the mechanism of induction of this cell death is not clear [26]. Earlier observations have shown that optineurin mediates interaction of TBC1D17, a GTPase activating protein, with Rab8 and regulates Rab8-mediated TFR trafficking and recycling. The E50K mutant causes defective recycling of TFR due to enhanced inactivation of Rab8 mediated by TBC1D17 [37]. Therefore, we explored the involvement of TBC1D17 in E50K-induced cell death by using a catalytically inactive mutant of TBC1D17 (R381A). This mutant of TBC1D17 has been shown to inhibit the function of endogenous TBC1D17 [37]. Coexpression of R381A mutant of TBC1D17 inhibited E50K-induced cell death whereas coexpression of wild type TBC1D17 did not (Figure 1A). Under these conditions, WT OPTN expressing cells showed 2–4% cell death similar to that obtained with GFP control transfection. Cell death inhibition in the presence of R381A was not due to its effect on E50K expression, as determined by western blotting (Figure 1B). E50K expression resulted in the activation of caspase-3, as measured by cleaved caspase-3 level in western blot (Figure 1B). Coexpression of R381A mutant but not wild type TBC1D17 with E50K mutant resulted in reduced level of cleaved caspase-3 (Figure 1B). These results suggest that TBC1D17, through its catalytic activity, mediates E50K-induced cell death. To further validate the role of TBC1D17 in E50K-induced cell death, we knocked down TBC1D17 by using shRNAs. The efficacy of these shRNAs to knockdown endogenous TBC1D17 was established by western blotting using antibody for TBC1D17 (Figure 1C). Knockdown of endogenous TBC1D17 by two different shRNAs resulted in reduced cell death and reduced level of cleaved caspase-3 induced by E50K expression (Figure 1C and D). These results provide further support for the suggestion that TBC1D17 mediates E50K-induced cell death. We next determined the colocalization of E50K-optineurin with TBC1D17. The cells grown on coverslips were transfected with GFP-OPTN or its E50K mutant, stained with TBC1D17 antibody and examined by confocal microscopy. Endogenous TBC1D17 showed diffuse cytoplasmic staining but in E50K expressing cells it was sequestered in vesicle-like structures formed by the E50K mutant (Figure 1D). The E50K mutant showed strong colocalization with endogenous TBC1D17 in these structures. Quantitative analysis of colocalization was carried out by calculating correlation coefficient. This analysis showed that TBC1D17 showed significantly better colocalization with the E50K mutant than with wild type optineurin (Figure 1E). Overexpressed TBC1D17 also showed significantly better colocalization with E50K mutant than wild type optineurin (data not shown).

**Figure 2:**
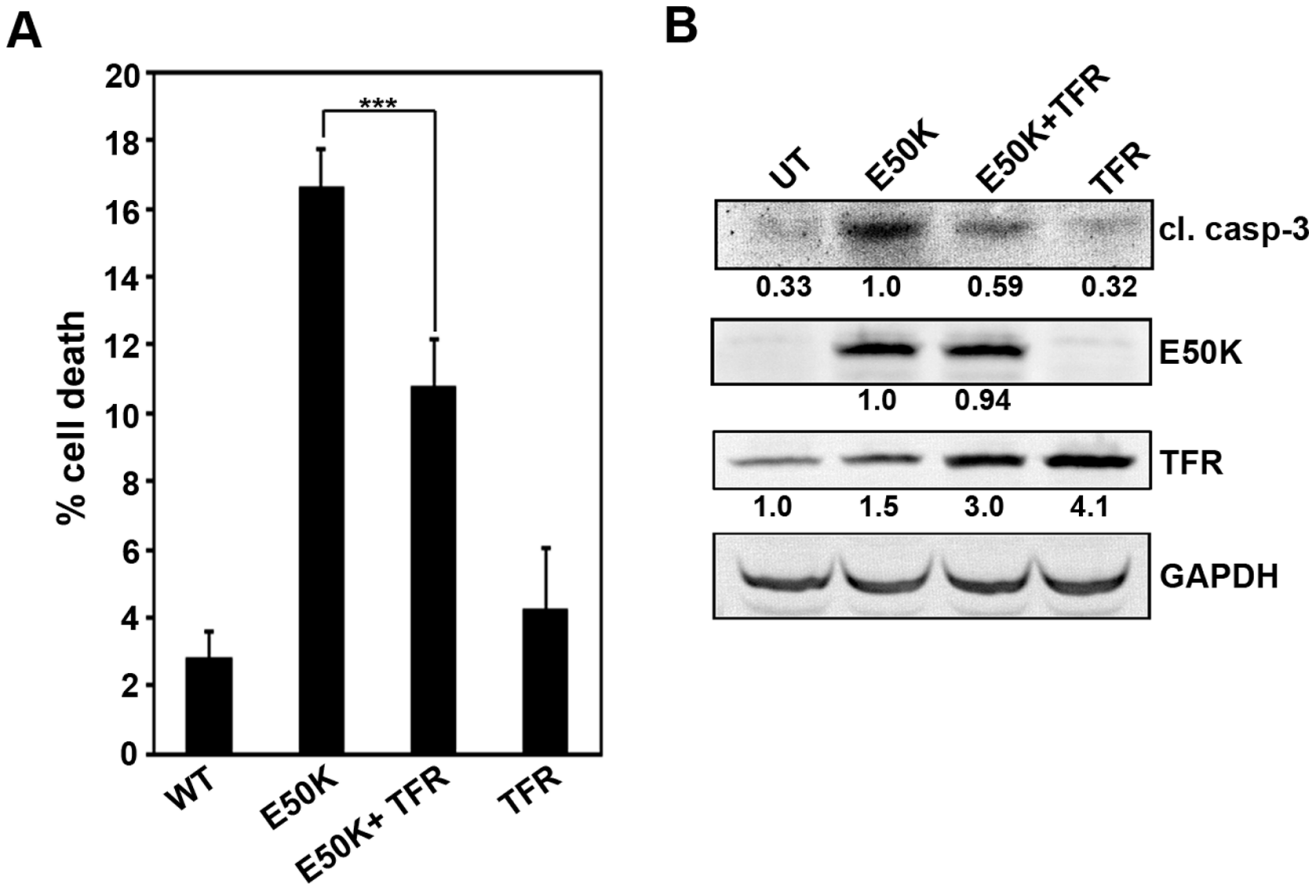
Effect of TFR expression on E50K-OPTN-induced cell death. (A) The cells plated on coverslips were transfected with 100ng of GFP-E50K alone or 200ng of TFR alone or cotransfected with 100ng GFP-E50K and 200ng TFR. After 32 hours of overexpression, cells were stained with TFR antibody and cell death was analysed. Data shown here represent cell death in expressing cells after subtracting background cell death from non-expressing cells from 6 experiments (mean±SD). ***p<0.001 (student’s t-test) (B) Cells plated in 35-mm dishes were transfected with 1 µg of GFP-E50K alone or 2 µg of TFR alone or cotransfected with 1 µg GFP-E50K and 2 µg TFR. After 32 hours of overexpression, cell lysates were made and subjected to western blotting with cleaved caspase-3, GFP and TFR antibodies. GAPDH was used as a loading control. UT, untransfected.

The E50K mutant impairs TFR recycling that is mediated by TBC1D17 [8], [37]. The impaired recycling of TFR could possibly contribute to E50K-induced cell death. Coexpression of TFR resulted in significant reduction of cell death induced by E50K-optineurin (Figure 2A). Also, coexpression of TFR with E50K showed decrease in cleaved caspase-3 level (Figure 2B). These results suggest that impaired TFR function partly contributes to E50K-induced cell death, but may not be a major mechanism contributing to this cell death.

### E50K-optineurin Inhibits Autophagy

**Figure 3:**
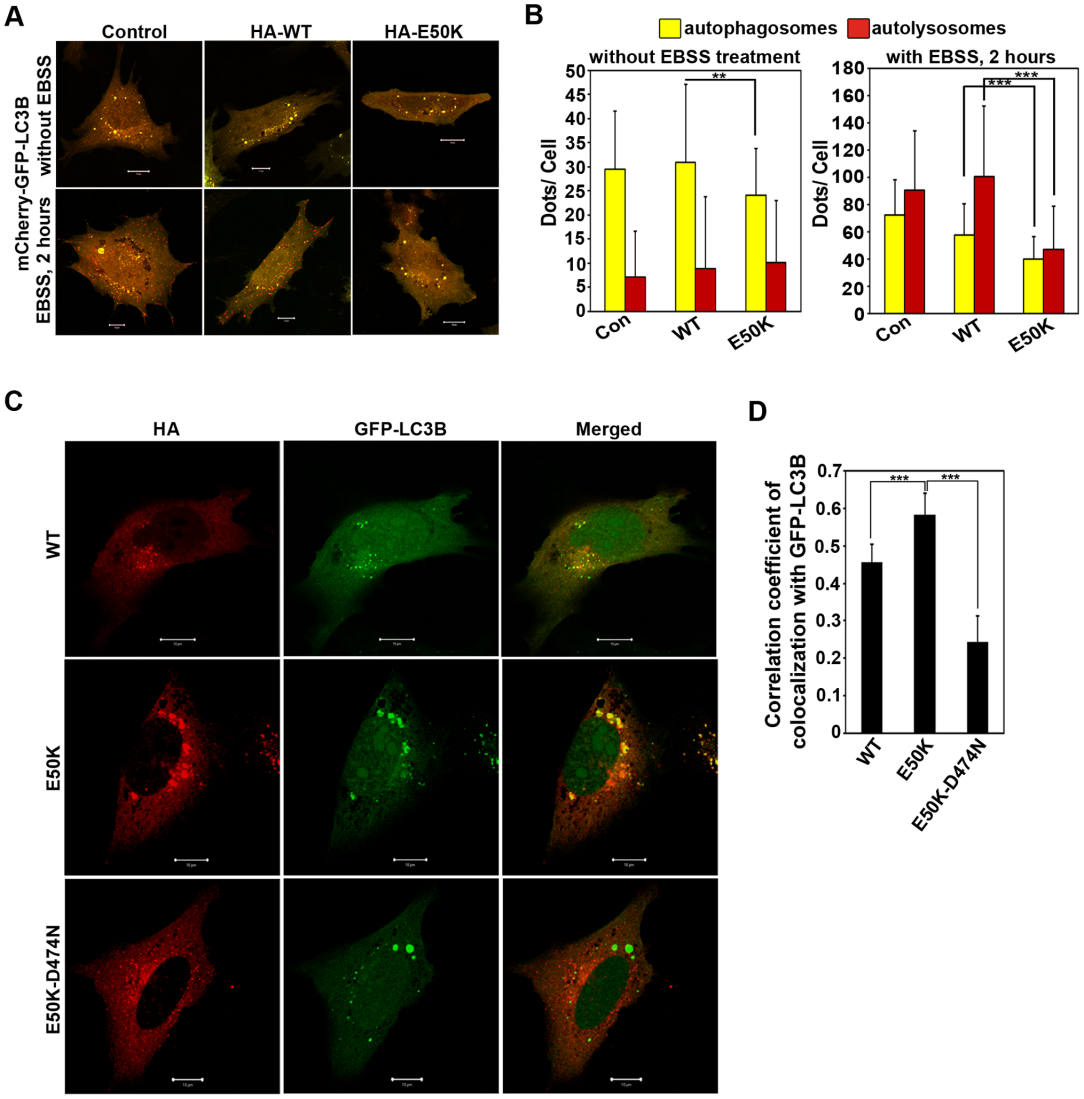
Inhibition of autophagic flux by E50K-OPTN. A,B. The cells plated on coverslips were cotransfected with 150ng mCherry-GFP-LC3B along with 150ng control plasmid (con) or HA-WT OPTN or HA-E50K plasmids. After 22 hours of transfection, cells were treated with aminoacid and serum free medium, EBSS for 2 hours. Images show representative fields with autophagosomes (yellow) and autolysosomes (red). Scale bar: 10 µm (A). Bar diagram showing number of autophagosomes (green) and autolysosomes (red) per cell (mean±SD) (B); n = 80 cells from 3 independent experiments. ***p<0.001 (C) The cells plated on coverslips were cotransfected with 150ng each of GFP-LC3B and HA-WT OPTN or HA-E50K or HA-E50K-D474N. After 24 hours, cells were fixed and stained with HA antibody. Images show colocalization of GFP-LC3B with various optineurin constructs by merging of green (LC3B) with red (optineurin constructs) showing yellow colour in merged panels. Scale bar: 10 µm (D) Bar diagram showing correlation coefficient of colocalization of optineurin or its mutants with GFP-LC3B. n = 20 cells. ***p<0.001.

Optineurin functions as an autophagy receptor through its ability to interact with LC3 (a protein present in autophagosomes) and ubiquitin [9]. Recently, we have shown that a glaucoma-associated variant of optineurin, M98K, causes enhanced autophagy leading to TFR degradation and death of retinal cells [10]. However, unlike M98K variant, the E50K mutant did not cause any reduction in TFR level; instead there was a small increase in TFR level in E50K expressing cells (as also seen in Figure 2B). This increase in TFR level in E50K expressing cells may be due to inhibition of autophagy because endogenous TFR in these cells is degraded by autophagy [10]. Therefore, we examined the possibility of impairment of autophagy by E50K mutation. For this purpose we measured the effect of E50K on autophagy flux and autophagosome formation by using mCherry-GFP-LC3B reporter construct, which goes to autophagosomes as well as autolysosomes, but the acidic pH of autolysosomes quenches the GFP signal and hence only red fluorescence is detected in the autolysosomes, whereas in autophagosomes both red and green fluorescence can be observed [50]. Autophagy flux was studied under both basal and aminoacid starvation (EBSS treated) conditions in WT OPTN and E50K expressing cells. Under basal conditions, expression of E50K-optineurin showed a decrease in autophagosome formation compared to WT OPTN (Figure 3A and B) while there was no change in flux (as measured by the number of autolysosomes). Induction of autophagy by amino acid starvation resulted in increased formation of autophagosomes and autolysosomes. Expression of E50K-optineurin, but not wild type optineurin, inhibited autophagy flux induced by amino acid starvation (Figure 3A and B). The number of autophagosomes was also reduced in E50K-expressing cells (Figure 3A and B) upon induction of autophagy by amino acid starvation. These results suggest that E50K-optineurin causes a defect in autophagy.

Previously, it has been shown that the E50K mutant forms large vesicular structures which are mostly positive for transferrin receptor [8], [28]. Many of these vesicle-like structures were identified as autophagosomes by colocalization with autophagosome marker, LC3 (Figure 3C and D). As compared to wild type OPTN the E50K mutant showed better colocalization with autophagosomes (GFP-LC3 positive vesicle-like structures) (Figure 3C and D). The ubiquitin binding defective mutant of E50K (E50K-D474N) showed much less localization to autophagosomes (Figure 3C and D). This suggests that localization of E50K-optineurin to autophagosomes requires its ubiquitin binding function.

**Figure 4:**
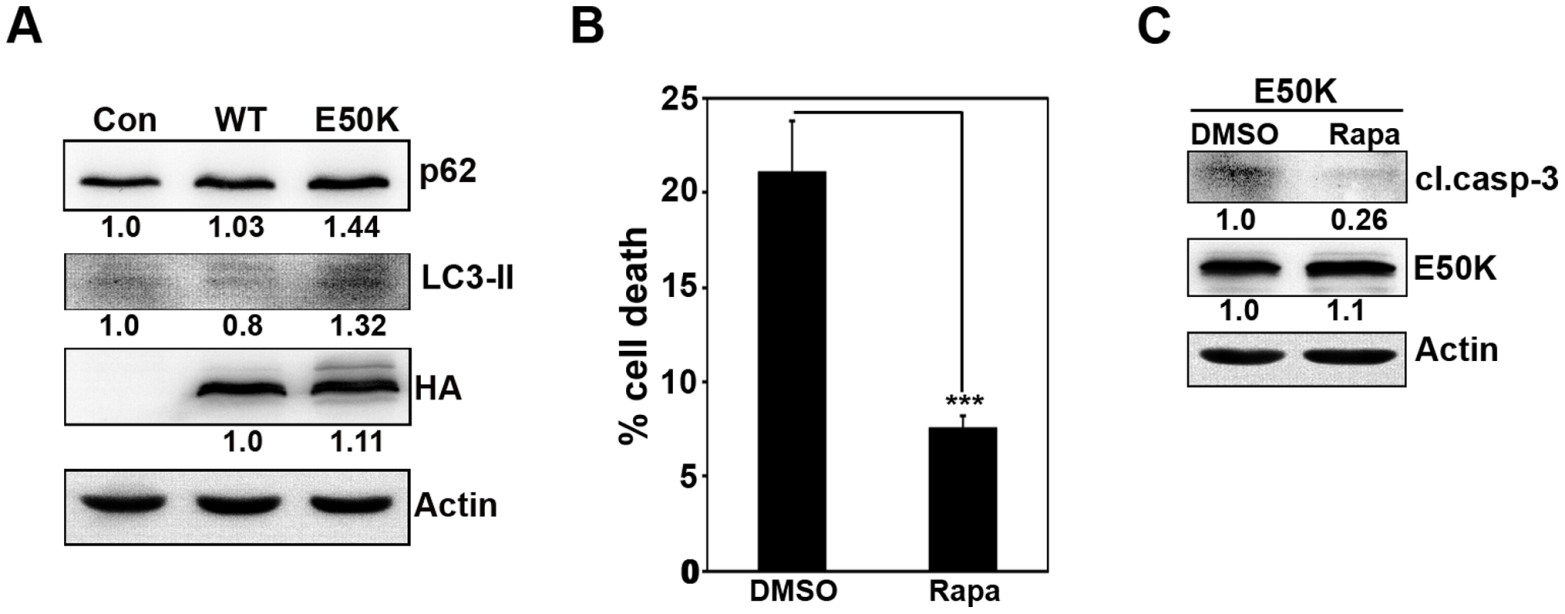
Involvement of autophagy in E50K-induced cell death. (A) Cells plated in 24-well plate were infected with control or WT OPTN or E50K adenoviruses and allowed to express for 24 hours. Protein lysates were subjected to western blotting with p62, LC3-II and HA antibodies. Actin was used as a loading control. (B) Cells plated on coverslips were transfected with GFP-E50K plasmids. After 12 hours of transfection, cells were treated either with DMSO or with 1 µM rapamycin (Rapa) for 20 hours. At the end of 32 hours of overexpression, cells were fixed and mounted to score for cell death. Bar diagram depicts percentage of cells showing E50K-induced cell death in the presence and absence of rapamycin. Data represents mean ± SD of 5 experiments. ***p<0.001 (Student’s t-test). (C) Cells plated in 35-mm dishes were transfected with GFP-E50K and after 12 hours of transfection, cells were treated with either DMSO or rapamycin (Rapa) (1 µM) for 20 hours. At the end of 32 hours of overexpression, lysates were made and subjected to western blot with cleaved caspase-3 and GFP antibodies. Actin was used as a loading control.

LC3, an autophagosome marker, exists in two forms where the cytosolic LC3-I form converts to lipidated LC3-II form which gets inserted into the autophagosomal membranes. The changes in the level of the LC3-II form is correlated with the degree of autophagosome formation and is used as an indicator to study changes in autophagy levels [51]. Apart from LC3, another substrate which can be used to study autophagy flux is P62/SQSTM1 (Sequestosome-1) [51]. This protein is normally degraded by autophagy and the accumulation of this protein is observed in response to autophagy inhibition. Hence, p62 is generally used as a marker to study inhibition of autophagy [51], [52]. Expression of E50K-OPTN but not wild type OPTN increased p62 and LC3-II levels in RGC-5 cells (Figure 4A). Moreover, treatment of E50K transfected cells with rapamycin, an inducer of autophagy, inhibited E50K-induced cell death significantly (Figure 4B), and also showed decrease in cleaved caspase-3 levels compared to DMSO treated E50K transfected cells (Figure 4C). These results suggest that E50K mutant of optineurin blocks autophagy and this block in autophagy mediates cell death.

**Figure 5:**
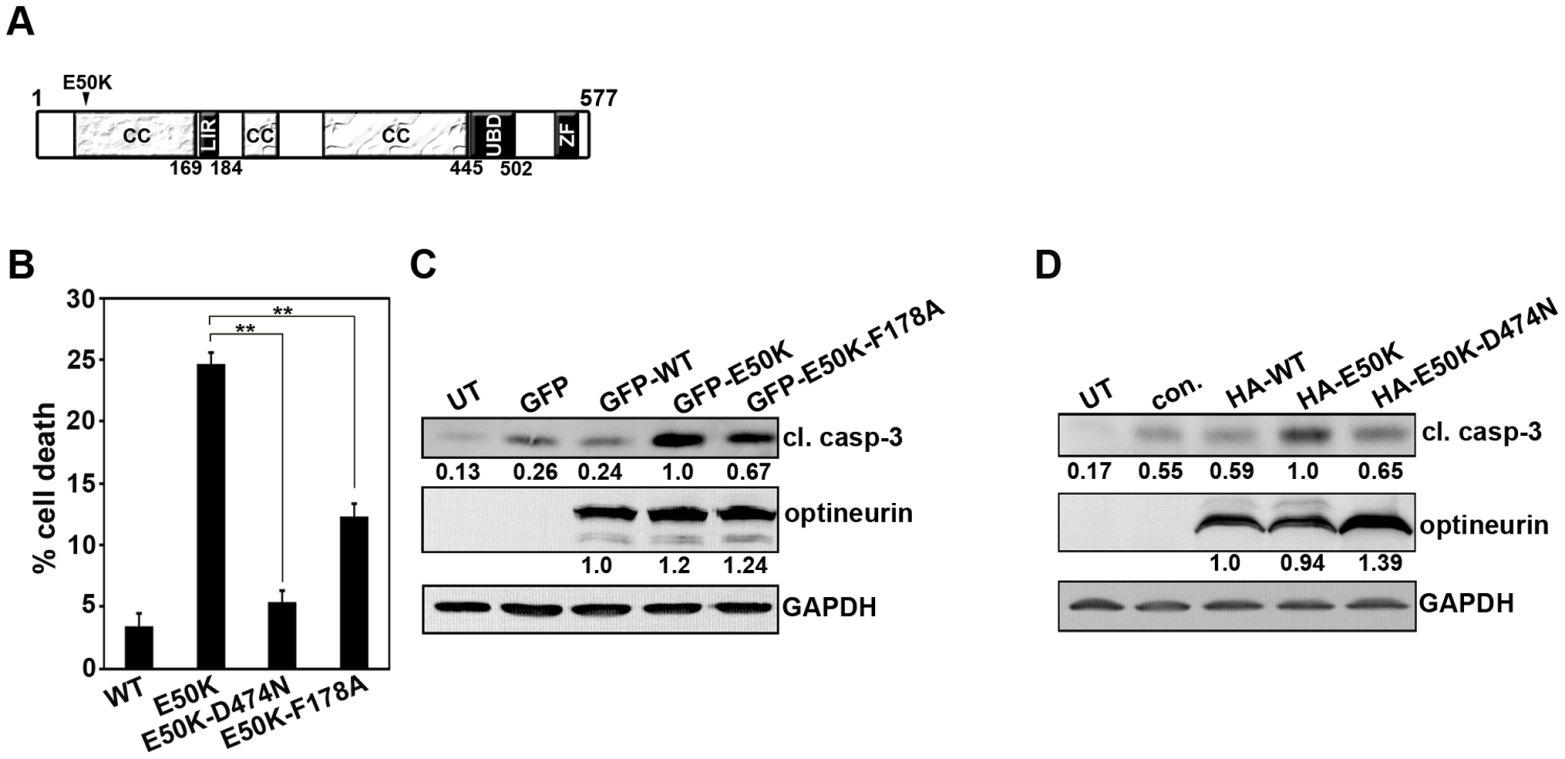
E50K-OPTN-induced cell death is dependent on UBD and LC3-interacting region. (A) Schematic of optineurin protein showing domains LIR (LC3 interacting region) and UBD (ubiquitin binding domain), ZF (zinc finger) and CC (coiled coil). (B) Cells plated on coverslips were transfected with 300ng of GFP-E50K or GFP-E50K-D474N or GFP-E50K-F178A. After 32 hours of transfection, cells were fixed and cell death was determined in the respective expressing cells. Bar diagram showing quantitation of cell death in the presence of each of the mutants after subtracting background cell death in non-expressing cells. Data represent mean ± SD of 3 experiments. **p<0.01 (Student’s t-test) (C) Cells were transfected with 3 µg of GFP control plasmid or GFP-WT or GFP-E50K or GFP-E50K-F178A plasmids and overexpressed for 32 hours. Western blot was done with cleaved caspase-3 and GFP antibodies. GAPDH was used as a loading control. UT, untransfected (D) The cells were transfected with 3 µg of control plasmid or HA-WT or HA-E50K or HA-E50K-D474N plasmids and overexpressed for 32 hours. Western blot was done with cleaved caspase-3 and HA antibodies.

Function of optineurin in autophagy depends on its ability to interact with LC3 and ubiquitin through well defined sites (shown schematically in Figure 5A) [9]. Mutation of Phe178 in the LC3-binding site of optineurin abrogates its interaction with LC3 [9]. To determine the role of interaction with LC3 in E50K-induced cell death, a double mutant, E50K-F178A, was used. As compared to E50K mutant, the expression of this mutant (E50K-F178A) resulted in reduced cell death (Figure 5B) and reduced level of cleaved caspase-3 (Figure 5C). Similarly, the expression of E50K-optineurin defective in ubiquitin binding (E50K-D474N) also resulted in reduced cell death and reduced formation of cleaved caspase-3 (Figure 5B and D). These results suggest that the interaction of optineurin with LC3 and ubiquitin, which are required for its autophagic function, are also required for E50K-optineurin induced cell death.

### TBC1D17 Inhibits Autophagy

**Figure 6:**
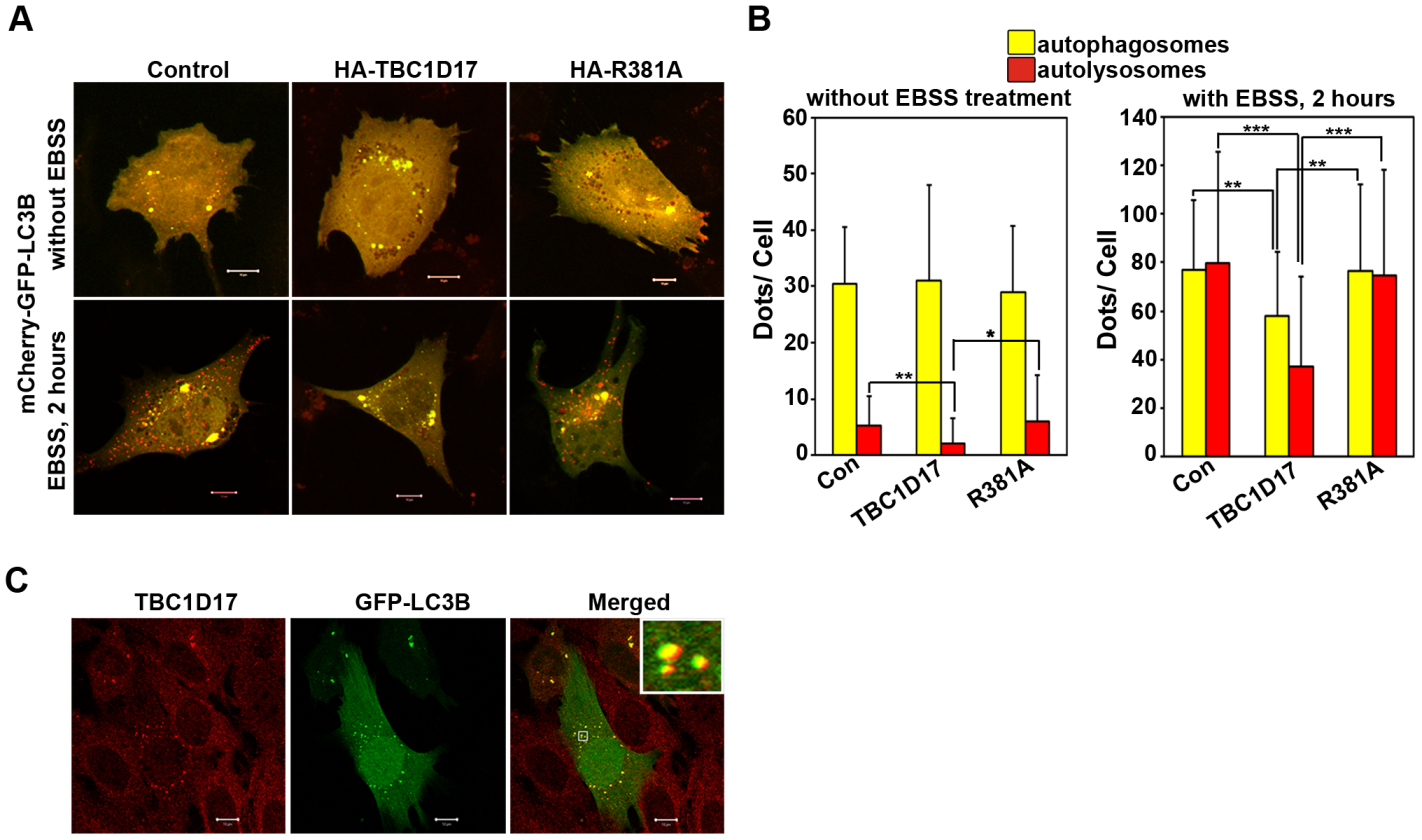
Inhibition of autophagy flux by TBC1D17. The cells plated on coverslips were cotransfected with 150ng of mCherry-GFP-LC3B along with 150ng of control plasmid or HA-TBC1D17 or HA- R381A plasmids. After 22 hours of transfection, one set of coverslips were kept untreated whereas the other set were treated with EBSS for 2 hours. (A) Images show representative fields with autophagosomes (yellow) and autolysosomes (red) Scale bar: 10 µm. (B) Bar diagram showing number of autophagosomes (yellow) and autolysosomes (red) per cell (mean±SD) n = 40 cells from 2 independent experiments. *p<0.05, **p<0.01, ***p<0.001 (C) The cells plated on coverslips were transfected with 100ng of GFP-LC3B and 22 hours after transfection stained with TBC1D17 antibody before confocal microscopy. Colocalization of TBC1D17 with LC3B in the foci is shown by merging of green (GFP-LC3B) with red (TBC1D17) giving yellow colour in the merged panels. Scale bar: 10 µm.

Our results showed that E50K-optineurin inhibits autophagy, and impaired autophagy contributes to E50K-induced cell death. Since TBC1D17, through its catalytic activity, mediates E50K-induced cell death, it raises the possibility of involvement of TBC1D17 in autophagy. To address the issue of role of TBC1D17 in autophagy we determined the effect of TBC1D17 on autophagy flux. Expression of TBC1D17, but not its catalytic mutant R381A, inhibited autophagy flux induced by amino acid starvation, as shown by a decrease in the number of autolysosomes (Figure 6A and B). The number of autophagosomes was also decreased in cells expressing TBC1D17 but not its R381A mutant (Figure 6A and B). Basal autophagy flux was also inhibited by TBC1D17 but not by its R381A mutant (Figure 6A and B). These results suggest that TBC1D17, through its catalytic activity, is involved in autophagy. Endogenous TBC1D17 showed colocalization with autophagosomes formed by GFP-LC3 (Figure 6C), further supporting the involvement of TBC1D17 in autophagy.

### TBC1D17 Mediates Inhibition of Autophagy Caused by E50K

**Figure 7:**
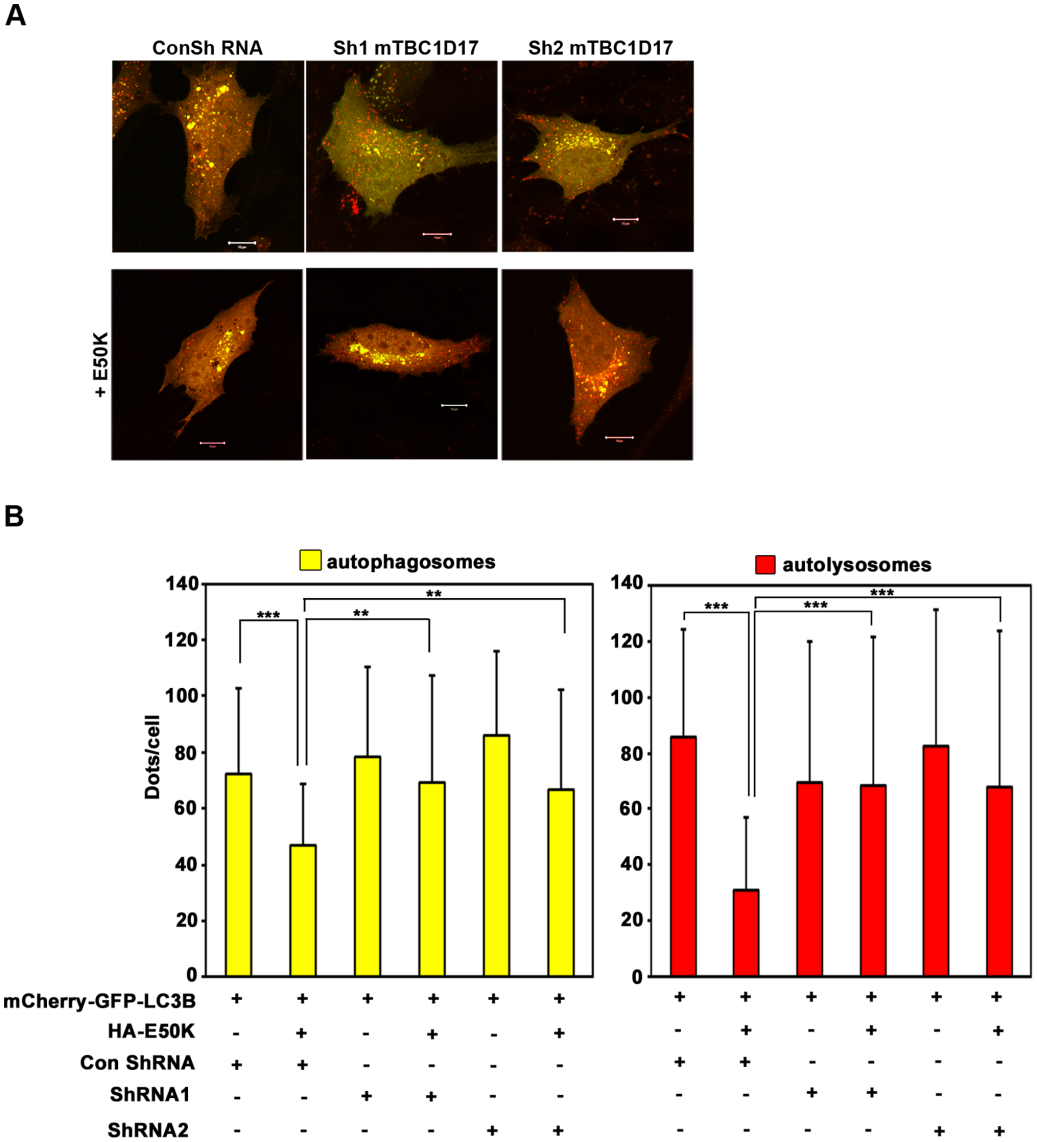
E50K-OPTN-induced inhibition of autophagic flux is rescued by TBC1D17 knockdown. The cells plated on coverslips were cotransfected with mCherry-GFP-LC3B and indicated plasmids. After 28 hours of transfection, cells were treated with EBSS for 2 hours. (A) Images showing representative fields with autophagosomes (yellow) and autolysosomes (red). Scale bar: 10 µm. Bar diagram showing number of autophagosomes (yellow) (B) and autolysosomes (red) (C) per cell in EBSS treated cells (mean±SD). n = 40 cells from 2 independent experiments. **p<0.01, ***p<0.001.

Our results presented so far showed that TBC1D17 has a role in E50K-induced cell death. Also, TBC1D17 overexpression inhibits autophagy. Since E50K-induced cell death involves autophagy, we hypothesized that E50K- dependent inhibition of autophagy may be mediated by TBC1D17. To test this hypothesis, we examined the role of TBC1D17 in E50K-dependent inhibition of autophagy flux induced by aminoacid starvation. Knockdown of TBC1D17 by two different shRNAs significantly resulted in reduced inhibition of autophagosome formation by E50K (Figure 7A and B). Inhibition of autolysosome formation by E50K was also reversed upon knockdown of TBC1D17 (Figure 7A and B). These results suggest that TBC1D17 mediates E50K-induced inhibition of autophagy.

Inhibition of autophagy by TBC1D17 requires its catalytic activity. Therefore, TBC1D17 is likely to inhibit autophagy by acting on one of the Rabs involved in autophagy. Recently, Rab12 has been shown to be involved in M98K-optineurin induced autophagy and autophagic cell death [10]. Overexpressed Rab12 colocalized with E50K vesicles and this colocalization was significantly better than that observed with wild type optineurin (Figure S1A and B). Endogenous TBC1D17 showed colocalization with Rab12 in E50K vesicles (Figure S1A) and this colocalization was significantly better in E50K vesicles as compared with that observed in wild type optineurin vesicles (Figure S1C). These observations raise the possibility that Rab12 may be involved in E50K-induced TBC1D17-dependent inhibition of autophagy.

## Discussion

Several mutations in optineurin have been identified that are associated with normal tension glaucoma [5], [20], [22], [23]. Only a few of these mutations are shown to alter functional properties of optineurin such as vesicle trafficking, NF-κB signaling and autophagy [8], [10], [14], [28]. The E50K mutation is the best studied mutation that was identified as a causative mutation because it segregated with disease phenotype in a large family [5]. E50K mutant causes death of retinal cells in cell culture model as well as transgenic mouse model, providing support for the suggestion that it causes the disease by directly inducing the death of retinal cells [26], [27]. Here, we have shown that E50K-induced death of retinal cells is mediated by a GTPase activating protein, TBC1D17. Our results show that TBC1D17, through its catalytic activity, inhibits autophagy. TBC1D17 also mediates E50K-induced inhibition of autophagy. Since E50K-induced cell death is inhibited by rapamycin (an inducer of autophagy), and requires interactions of E50K with LC3 and ubiquitin that are involved in autophagy, it is likely that E50K-induced inhibition of autophagy, mediated by TBC1D17, leads to cell death.

Optineurin was identified as an autophagy receptor. Function of optineurin in autophagy depends on its ability to bind directly with LC3 and ubiquitin through well defined binding sites [9]. Involvement of autophagy and proteasome in E50K-induced cell death has been suggested earlier but the mechanisms are not understood [31]. Our results show that mutation of LC3-binding site or UBD by point mutations reduced E50K-induced cell death suggesting that these interactions play an important role in E50K-induced cell death. The vesicle-like structures formed by E50K-OPTN expression were all positive for TFR. Many but not all the vesicles formed were also positive for the autophagosome marker, LC3, suggesting that these structures were formed partly due to inhibition of autophagy (Figure S2). Therefore, it is likely that the E50K foci, which are positive for TFR but not for LC3 are formed due to alternate mechanisms such as inhibition of vesicle trafficking (TFR recycling).

Although several glaucoma-associated mutants of optineurin have been tested to induce death of retinal cells, only E50K and M98K variants are able to induce more cell death than wild type optineurin [10], [26]. M98K polymorphism is associated with glaucoma in certain ethnic groups [10]. Like E50K mutant, M98K-OPTN induces cell death selectively in retinal cells but not in other cell lines tested [10]. But, unlike E50K-induced cell death, M98K-induced cell death is not inhibited by antioxidants or antiapoptotic protein Bcl2. Expression of M98K induces autophagy leading to transferrin receptor degradation and cell death. TFR degradation is not seen upon E50K expression indicating that the two variants signal to cell death by engaging different effectors. M98K-OPTN-induced cell death involves autophagy which can be prevented by knockdown of Atg5, a protein essential for autophagy, or by inhibition of autophagy by chemical inhibitors [10]. Transferrin receptor degradation by autophagy plays a crucial role in M98K-induced cell death as shown by nearly complete inhibition of M98K-induced cell death upon coexpression of TFR. Thus an appropriate level of autophagy is essential for survival of retinal cells because enhanced autophagy, as seen with M98K-OPTN, or a block in autophagy, as shown by E50K-OPTN, can lead to cell death.

**Figure 8:**
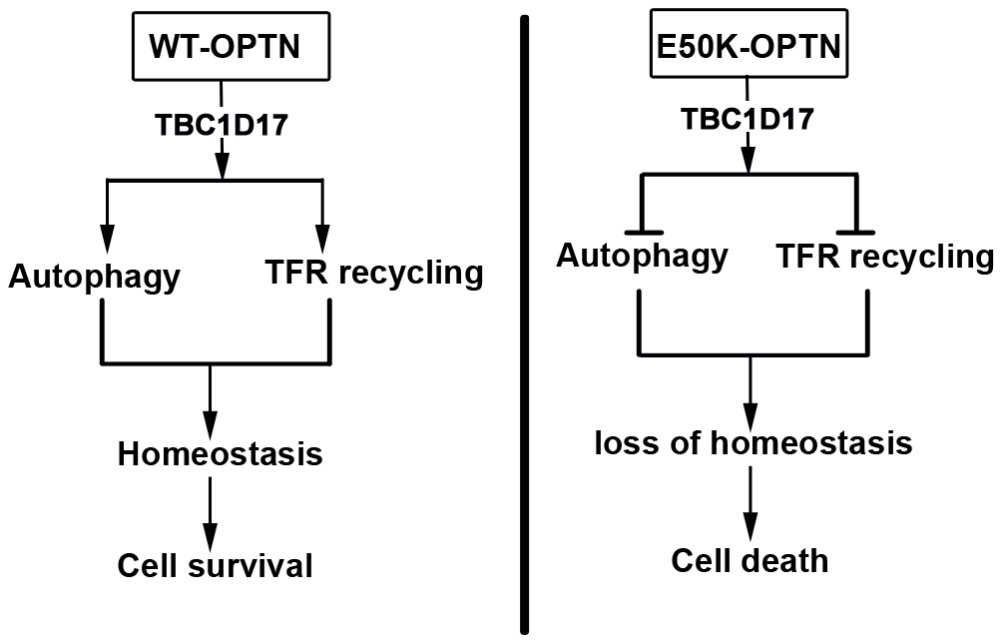
A model showing steps involved in E50K-OPTN-induced death of retinal cells. In cells expressing wild type optineurin, it plays a role in autophagy and also in TFR trafficking and recycling. This maintains homeostasis that is necessary for survival of normal cells. In retinal cells expressing E50K-OPTN, autophagosomes are formed but autophagy flux (autolysosome formation) is inhibited that leads to accumulation of large autophagosomes. TFR recycling is also impaired in E50K expressing cells. In both these processes TBC1D17 plays a crucial role. Impaired autophagy as well as impaired TFR recycling leads to loss of cellular homeostasis that results in cell death. This cell death can be largely prevented by blocking TBC1D17 function and partly by overexpressing TFR.

Our results suggest that TBC1D17, through its catalytic activity, mediates E50K-dependent inhibition of autophagy that leads to cell death. Previously we have shown that TBC1D17 mediates E50K-dependent inhibition of TFR recycling [37]. Results presented here suggest that impairment of TFR function partly contributes to E50K-induced cell death because coexpression of TFR reduced this cell death. Thus it appears that in E50K-induced cell death, TBC1D17 performs two different functions, inhibition of autophagy and inhibition of TFR recycling (Figure 8). Inhibition of TFR recycling by TBC1D17 is due to its ability to inhibit Rab8 [37]. However, it is not clear as to how TBC1D17 inhibits autophagy. Since catalytic activity is required for inhibition of autophagy by TBC1D17, it is likely to act on one of the Rab proteins involved in autophagy. Whether TBC1D17 acts on Rab12 that is involved in EBSS or M98K-optineurin induced autophagy and also forms a complex with optineurin [10], is yet to be investigated. Some indication for the involvement of Rab12 in E50K-induced inhibition of autophagy is provided by the observations that Rab12 colocalizes with E50K vesicles and this colocalization is better than that observed with wild type optineurin. In addition, Rab12 shows colocalization with TBC1D17 in E50K vesicles. Rab7, another Rab that is involved in autophagy, shows some colocalization with E50K [8], [53]; therefore, it might be involved in E50K mediated inhibition of autophagy. Although Rab8 is not known to be involved in autophagy, we cannot rule out its involvement in E50K mediated inhibition of autophagy.

## Conclusions

Overall our results suggest that TBC1D17, a GAP for Rab GTPases plays an important role in E50K-induced death of retinal cells. Interactions of optineurin with LC3 and ubiquitin that are crucial for autophagic function are important for induction of cell death by E50K. TBC1D17 mediates E50K-induced inhibition of autophagy leading to formation of large autophagosomes and cell death. E50K-induced inhibition of TFR function also contributes to cell death.

## Supporting Information

Figure S1 Colocalization of OPTN/E50K with Rab12. The cells plated on coverslips were transfected with GFP-RAB12 and HA-WT-OPTN or E50K mutant and overexpressed for 24 hours. Cells were then stained with HA and TBC1D17 antibodies. (A) Colocalization of Rab12 (green), WT OPTN/E50K (blue) and endogenous TBC1D17 (red) is shown in merged panels. Scale bar: 10 µm. (B) Bar diagram showing correlation coefficient of colocalization of Rab12 with WT-OPTN and E50K mutant. (C) Bar diagram showing correlation coefficient of colocalization of Rab12 with endogenous TBC1D17 in cells expressing WT-OPTN and E50K mutant. n = 15 cells. *** p<0.001
(TIF)

Figure S2 Colocalization of optineurin and TFR with autophagosomes. Cells plated on cover slips were transfected with GFP-LC3 and HA-WT/E50K and stained with HA (blue) and TFR (red) antibodies. Images show TFR colocalization with GFP-LC3B structures (autophagosomes) in WT or E50K expressing cells. Scale bar: 10 µm
(TIF)
