# Supplementary figures and images for: E50K-OPTN-Induced Retinal Cell Death Involves the Rab GTPase-Activating Protein, TBC1D17 Mediated Block in Autophagy
Source: PLoS One. 2014 Apr 21;9(4):e95758. doi: 10.1371/journal.pone.0095758 (PMC3994150; doi:10.1371/journal.pone.0095758)

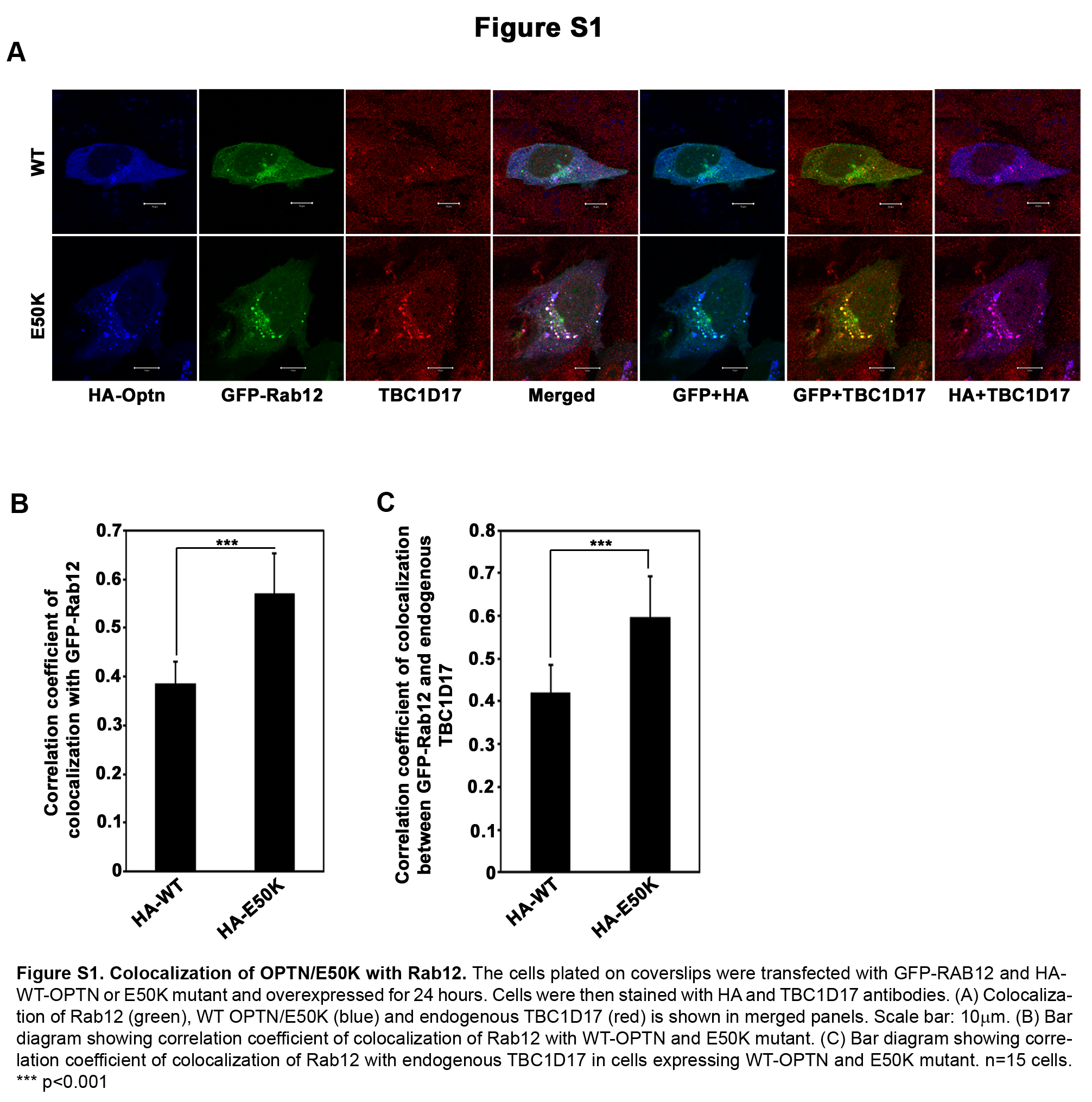

Supplement: Figure S1 — Colocalization of OPTN/E50K with Rab12. The cells plated on coverslips were transfected with GFP-RAB12 and HA-WT-OPTN or E50K mutant and overexpressed for 24 hours. Cells were then stained with HA and TBC1D17 antibodies. (A) Colocalization of Rab12 (green), WT OPTN/E50K (blue) and endogenous TBC1D17 (red) is shown in merged panels. Scale bar: 10 µm. (B) Bar diagram showing correlation coefficient of colocalization of Rab12 with WT-OPTN and E50K mutant. (C) Bar diagram showing correlation coefficient of colocalization of Rab12 with endogenous TBC1D17 in cells expressing WT-OPTN and E50K mutant. n = 15 cells. *** p<0.001 (TIF) [file pone.0095758.s001.tif]

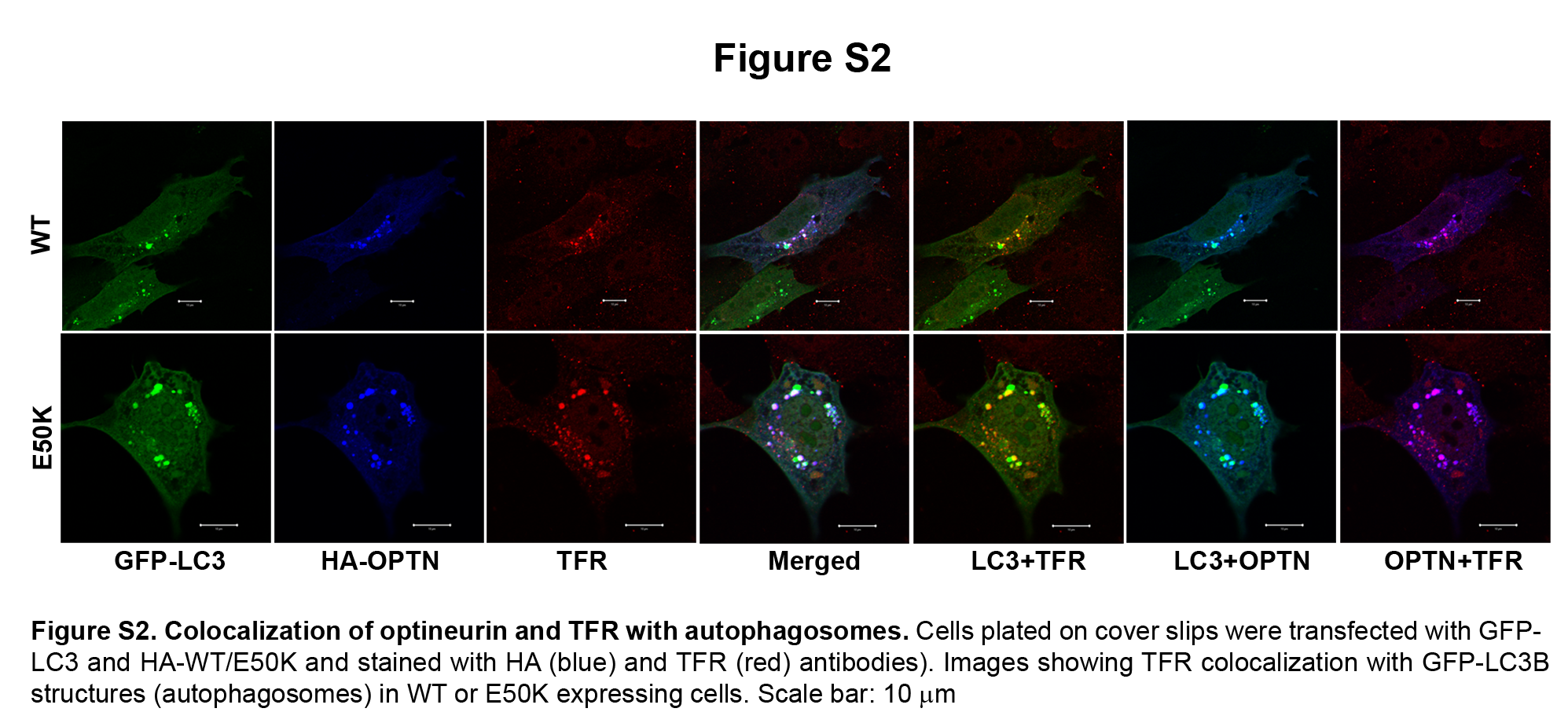

Supplement: Figure S2 — Colocalization of optineurin and TFR with autophagosomes. Cells plated on cover slips were transfected with GFP-LC3 and HA-WT/E50K and stained with HA (blue) and TFR (red) antibodies. Images show TFR colocalization with GFP-LC3B structures (autophagosomes) in WT or E50K expressing cells. Scale bar: 10 µm (TIF) [file pone.0095758.s002.tif]
